# Cardiovascular Autonomic Neuropathy in Context of Other Complications of Type 2 Diabetes Mellitus

**DOI:** 10.1155/2013/507216

**Published:** 2013-12-18

**Authors:** Anca Moţăţăianu, Rodica Bălaşa, Septimiu Voidăzan, Zoltán Bajkó

**Affiliations:** ^1^Department of Neurology, University of Medicine and Pharmacy Târgu Mureş, Târgu Mureş, Gheorghe Marinescu, No. 38, 540139, Romania; ^2^Mureş County Clinical Emergency Hospital, Târgu Mureş, Gheorghe Marinescu, No. 50, 540136, Romania; ^3^Department of Epidemiology, University of Medicine and Pharmacy Târgu Mureş, Târgu Mureş, Gheorghe Marinescu, No. 38, 540139, Romania

## Abstract

The aim of this study was to investigate the relationship between cardiac autonomic neuropathy (CAN) and other micro- and macrovascular complications and risk factors for type 2 diabetes. We included, in this study, 149 patients with type 2 diabetes. We evaluated their cardiovascular risk factors, demographic data, and any major micro- and macrovascular complications of their diabetes. Assessments of CAN were based upon Ewing's battery. *Results*. CAN was present in 38.9% of patients. In the CAN group, the duration of diabetes, BMI, systolic blood pressure, lipid levels, and HBA1c were all significantly higher than those in the other group. A significant association was found between CAN and retinopathy, peripheral neuropathy, ABI, and IMT. Multivariate logistic regression demonstrated that, in type 2 diabetes, the odds of CAN (OR (95% confidence intervals)) increase with the age of the patients (1.68 (1,4129–2.0025)), the average diabetes duration (0.57 (0.47–0.67)), cholesterol (1.009 (1.00-1.01)), HbA1c levels (1.88 (1.31–2.72)), peripheral neuropathy (15.47 (5.16–46.38)), BMI (1.12 (1.05–1.21)), and smoking (2.21 (1.08–4.53)). *Conclusions*. This study shows that CAN in type 2 diabetes is significantly associated with other macro- and microvascular complications and that there are important modifiable risk factors for its development.

## 1. Introduction 

Among chronic diabetic complications, cardiac autonomic neuropathy (CAN) is one of the most common, but it is also one of the most frequently ignored. Currently, a general consensus exists that CAN is an independent risk factor for cardiovascular events [1, 2]. Its high mortality rate is related to cardiac arrhythmias, silent myocardial ischemia, sudden death, perioperative cardiovascular, and cardiorespiratory instability [3].

The American Diabetes Association (ADA) considers cardiovascular reflex tests to be the appropriate diagnostic tests for CAN because they have good sensitivity, specificity, and reproducibility, and they are easily performed [4].

CAN assessment may be used for cardiovascular risk stratification in patients with and without established cardiovascular disease, as a marker for patients requiring more intensive pharmacotherapeutic and life-style management of comorbid conditions [4].

Long durations of metabolic disturbances can cause vascular damage, leading to both micro- and macrovascular complications. Conclusive clinical evidence from a large prospective clinical study showed that hyperglycaemia plays a central role in the pathogenesis of CAN, although other metabolic and vascular factors contribute to the disease state and its progression [5, 6]. Hyperglycaemia also seems to play an important role in the pathogenesis of diabetic macrovascular disease [7].

The Steno2 study demonstrated the clinical importance of multifactorial interventions to improve cardiovascular outcomes in patients with type 2 diabetes [8]. Thus, understanding the risk factors for CAN and detecting subclinical CAN early on are crucially important for treatment and for preventing potentially serious consequences of CAN.

A close association between CAN and other diabetic microvascular complications, such as retinopathy, nephropathy, and peripheral neuropathy, has been observed in previous studies, possibly related to changes in the vasomotor control of small vessels [9, 10].

Ewing's battery is currently the gold standard in clinical autonomic testing, but it requires patient cooperation and use of normal age-related values. New methods that are noninvasive and independent of patient cooperation, as spectral heart rate variability and ambulatory blood pressure monitoring, offers early additional information and risk stratification and can be used for clinical diagnosis of CAN. Other methods, like evaluation of baroreflex sensitivity, cardiac radionuclide imaging, or invasive microneurography, have low availability and limited standardization, so they are not indicated for clinical diagnosis in routine daily practice but can be used in research perspectives [11].

The aim of this study was to examine the prevalence of CAN, the risk factors for CAN and the relationship between CAN, and other micro- and macrovascular complications in type 2 diabetic patients.

## 2. Methods 

### 2.1. Patients

In this prospective study, 164 consecutive patients with type 2 diabetes mellitus (age 20–65) were selected among all diabetic subjects who presented to the Diabetes Department of the University Hospital (Targu Mures, Romania). Study subjects met the American Diabetes Association criteria for type 2 diabetes. Exclusion criteria were as follows: presence of cardiac arrhythmia, heart block, clinical coronary artery disease, presence of thyroid disease (hypo- or hyperthyroidism), presence of hypo- or hyperglycaemia in the previous 24 h, presence of acute illness, severe systemic disease such as cardiac, pulmonary, or kidney insufficiency, medication that affects the autonomic nervous system (antiarrhythmic medication, antidepressants, antihistamine, and sympathomimetic cough preparations), advanced diabetic retinopathy, other obvious causes of neuropathy, for example, alcohol abuse, use of neurotoxic medication or malignant disease, history of diabetic ketoacidosis, and other secondary causes of diabetes [1]. Based upon these exclusion criteria, 15 patients were excluded from the study group.

This study protocol was approved by the University of Medicine Tg. Mures Review Board, and all participants gave their written informed consent.

### 2.2. Clinical and Metabolic Parameters Assessment

A standardised questionnaire was used to obtain medical histories for all patients (including diabetes duration, previous and current diseases, and use of medication) as well as lifestyle behaviour information (smoking habits, alcohol consumption, and physical activity).

All patients completed a questionnaire on neuropathic and autonomic symptoms, and their scores were used to identify subjective symptoms of motor, sensory, or autonomic neuropathy in the previous six months. All patients underwent a complete physical examination, including blood pressure, body mass index (BMI), and waist-to-hip ratio (WHR). A patient was classified as hypertensive if the systolic blood pressure (SBP) was ≥140 mmHg or if the diastolic blood pressure (DBP) was ≤90 mmHg or if the patient used any antihypertensive medications.

Blood samples were collected in the morning after an overnight fast. Fasting plasma glucose (FPG), total cholesterol, triglycerides, and creatinine levels were measured with an automatic analyser. Glycosylated hemoglobin (HbA1c) was determined by high-performance liquid chromatography, with a nondiabetic reference range of 4.1–6.0. Estimated glomerular filtration rate (eGFR) was calculated using the Modification of Diet in Renal Disease study (MDRD) equation [12].

Diabetic retinopathy was evaluated by an experienced independent ophthalmologist. Direct fundoscopy was performed on dilated pupils, and the findings were classified as normal, preproliferative retinopathy (including maculopathy), or proliferative retinopathy.

### 2.3. Peripheral Neuropathy Assessment

Neuropathic symptoms were assessed based upon neuropathy symptom scores as previously described [13]. All patients underwent clinical neurological examination, quantified by the neuropathic disability score (NDS). Reflexes were graded at the knee and ankle on a scale with a maximum of eight points if the joints were areflexic. Sensory tests include pinprick sensation, light touch, and vibration and temperature perception. A score was given according to the anatomic location at which the patient could identify the introduced stimulus. If the patient perceived the stimulus at all levels, a score of 0 was given. A score of 1 was given if the patient failed to perceive the stimulus at the base of the toe, 2 if the patient failed to perceive the stimulus at the mid-foot, 3 at the heel, 4 at the lower leg, and 5 if at or above the knee level. The average score for both feets was entered as the sensory score. An NDS score of 0–2 was defined as “no neuropathy,” and scores ≥3 were considered to indicate neuropathy [14].

An electrodiagnostic protocol as recommended by the ADA was used for nerve conduction studies (NCS) [15]. For each patient, standard nerve conduction measurements were performed on the median, ulnar, peroneal, tibial, and sural nerves in both the upper and lower extremities, according to standard techniques [16]. NCS were performed according to conventional methods with a four-channel neuro-MEP electromyography with surface electrodes in a room with a constant temperature between 22 and 24°C. Skin temperatures of the arm and leg were between 32 and 36°C. All electrophysiological tests were performed by the same examiner. The reference values were obtained from laboratory controls consisting of 50 healthy subjects aged 20–65 years.

Motor nerve conduction velocities (MCV), compound muscle action potential amplitudes (CMAP), and distal motor latencies (DML) were determined bilaterally in the motor nerves. Sensory nerve conduction velocities (SCV), sensory nerve action potential amplitudes (SNAP), and distal sensory latencies (DSL) were determined bilaterally in the sensory nerves. The amplitudes of the motor and sensory responses were measured to the first negative peak. Slowness in the MCV or SCV less than the normal limit (mean − 2 SD), reduced amplitudes in the CMAP or SNAP less than the normal (mean − 2 SD), or prolonged latencies in the DML or DSL more than normal limit (mean + 2 SD) were identified as abnormal values [16]. When two or more nerves were abnormal, NCS were considered abnormal according to the Mayo Clinic staging criteria [17].

The patients were classified as having subclinical peripheral neuropathy in the absence of signs or symptoms of neuropathy if they had abnormal NCS. They were classified as having confirmed peripheral neuropathy in the presence of abnormal NCS and symptoms or signs of neuropathy, and they were considered to have no peripheral neuropathy if NCS were normal with no symptoms or signs of neuropathy on clinical examination [4].

### 2.4. Cardiovascular Autonomic Neuropathy Assessment

Patients were requested to avoid strenuous physical exercise in the 24 h preceding the cardiovascular testing and to avoid smoking, eating, or coffee consumption for at least 2 h prior to testing. All antidiabetic and other medications were administered at the end of the examination.

Cardiovascular autonomic reflex tests were performed by one examiner early in the morning according to Ewing's method, which includes a battery of five noninvasive autonomic tests [18]. Parasympathetic functions were analysed based upon the heart rate response to slow deep breathing, to Valsalva manoeuvring, and to a postural change from lying to standing.Heart rate responses were assessed from electrocardiographic recordings of R-R intervals automatically using an ELI 250 electrocardiograph system (Research Technology Inc.). These tests were performed using technique-specific normative data, as previously described [11]. The test results of the deep-breathing test were interpreted according to normal age-related values [19]. Sympathetic function was assessed by measuring blood pressure response to postural change from lying to standing and to sustained handgrip. Details of these assessments of cardiovascular autonomic function have been previously described [1].

The diagnostic criteria obtained by applying Ewing's five standard tests are summarised in Table 1. These values disregard age-specific differences in the tested functions as previously mentioned.

Cardiovascular autonomic neuropathy (CAN) was defined as the presence of at least 2 abnormal standard tests [4].

### 2.5. Macroangiopathic Complication Assessment

The carotid intima-media thickness (IMT) was assessed by one trained physician using ultrasonography (Siemens Acuson Antares Ultrasound System) on both bilateral common carotid arteries with a linear array 5 mHz transducer as reported previously [20]. Scanning was performed at three different longitudinal projection sites (anterior-oblique, lateral and posterior-oblique). The IMT was measured at the thickest portion of the scanning area and at two other points, 1 cm upstream, and 1 cm downstream from the site of greatest thickness. The mean of these three IMT measurements was used as the individual's IMT. We also evaluated the ankle-brachial index (ABI) with a handheld 5 mHz Doppler device (HI Dop Vascular Doppler set) in all patients.

### 2.6. Statistical Analyses

Statistical analyses were performed using MedCalc Software (Version 12.3.0 bvba, Mariakerke, Belgium). Student's *t*-tests were used to assess differences between continuous variables (expressed as mean ± SD), and  *χ* ^2^  tests were used for categorical variables (expressed as number and percentage). Differences between nonparametric variables (expressed as median, range) were compared using Mann-Whitney  *U*  tests. We applied univariate logistic regression and multivariate logistic regression. Once we had developed models for comparison, multivariate logistic regression analyses were used to evaluate independent associations between CAN and variables of interest. Statistical analyses were performed by calculation of the odds ratio (OR). We considered the results statistically significant when *P* < 0.05 and when the 95% confidence interval (CI) did not include the value 1. A significance level of 0.05 was used for all analyses, and all  *P*  values reported are two tailed.

## 3. Results

### 3.1. Comparison between Type 2 Diabetic Patients with and without CAN

Patients with all 5 autonomic tests classified as normal and those with one abnormal autonomic test were classified as “without CAN” (CAN−). Those patients with two or more abnormal tests were classified as “with CAN” (CAN+). Additional clinical characteristics and laboratory findings stratified by the presence of CAN are shown in Table 2. Compared to the patients without CAN, patients with CAN were younger at the time of diagnosis of diabetes (*P* = 0.0001) and had longer diabetes durations (CAN− median 4 (range 0–15) versus CAN+ median 12.5 (5–37); *P* = 0.0001), poorer glycaemic control as indexed by HbA1c levels (*P* = 0.0001), poorer lipid profiles (*P* = 0.001), lower eGFR (*P* = 0.0001), significantly higher SBP values (*P* = 0.04), and were more frequently smokers (*P* = 0.02). Patients with CAN had more retinopathy, peripheral neuropathy, increased IMT, increased QTc, and lower ABI than those without CAN. Patients with CAN had higher BMI (*P* = 0.0001) and a central distribution of fat reflected by their waist-hip ratios (WHR) in both genders, but these measurements were more significant in female patients (*P* = 0.002 in female and *P* = 0.005 in male). No significant differences in age, sex, or diastolic blood pressure were found between the two groups.

Of the 58 patients with CAN, 7 (12.06%) were clinically asymptomatic, 24 (41.37%) complained of gastrointestinal symptoms, 7 (12.06%) of cardiovascular symptoms, 3 (5.17%) of urinary bladder dysfunction or impotence in men, 14 (24.13%) have combination of gastrointestinal and cardiovascular symptoms, and 3 (5.17%) have combination of gastrointestinal, cardiovascular, and genitourinary symptoms.

### 3.2. Logistic Regression Analysis

#### 3.2.1. CAN, Univariate Correlations

Univariate logistic regression analysis (Table 3) demonstrates that, in type 2 diabetic patients, the odds of CAN increased with longer duration of diabetes, higher BMI, increased SBP, poor glycaemic control, dyslipidaemia, the presence of smoking, the presence of retinopathy, peripheral neuropathy, and with higher IMT values. The prevalence of CAN increased steadily with increasing duration of diabetes (OR 1.6; IC: 1.4–1.9, *P* = 0.0001). The proportion of those patients treated with insulin was significantly higher in patients with CAN than in patients without CAN.

#### 3.2.2. Multivariate Analysis

Multiple logistic regression (Table 4) in model 1, with CAN as the dependent variable and 3 predictor variables (age, sex, and diabetes duration), showed that longer diabetes duration (OR 1.74; *P* < 0.0001) and patient age (OR 1.48; *P* < 0.0001) were associated with CAN but not with the sex of the patient.

In the multivariate analysis in model 2, CAN was significantly (dependent) associated with elevated levels of cholesterol (1.009; *P* = 0.03) and poor glycaemic control as indexed by HbA1c levels (1.88, *P* = 0.0007), but it was not associated with current FPG or triglyceride levels.

Multivariate analysis in model 3 showed a statistically significant association between CAN and peripheral neuropathy. The presence of peripheral neuropathy was associated with 15 times greater risk of developing CAN.

Multivariate analysis in model 4 revealed dependent associations between CAN, smoking, and BMI.

## 4. Discussion

The reported prevalence of CAN varies greatly depending upon the populations studied and the different diagnostic tools used. The prevalence of CAN in our patient group with type 2 diabetes was 38.9% (taking into account the 15 excluded patients based on the established exclusion criteria, the real prevalence was between 35.3 and 44.5%). This result is consistent with previous prevalence rates reported in other studies using similar diagnostic criteria [21, 22]. Our study demonstrated that CAN is a common complication in type 2 diabetes.

Diabetic microvascular complications are closely related to one another because there is a common pathophysiology for these microvascular complications. Dyck studied diabetic neuropathy in a prospective study over a period of 7 years and demonstrated that the strongest predictor for the development and progression of neuropathy was the severity of retinopathy and 24-hour proteinuria [23]. Thus, a clear epidemiological link exists between the development and progression of neuropathy and retinopathy and nephropathy.

Our study results reinforced the concurrent development of CAN and other microangiopathic complications (retinopathy and peripheral neuropathy). We found a link between increasing severity of CAN and increasing prevalence and severity of peripheral neuropathy and retinopathy, which are markers of microangiopathic complications. The present results are in accordance with previous studies that have reported that the presence of other microangiopathic complications is associated with higher prevalence and early onset of CAN [10, 22, 24].

A coexistence between somatic and autonomic neuropathies was found in our study, and a significant statistical correlation was found between the degree of autonomic and somatic nervous system impairment. Our findings support the parallel development of somatic and autonomic nerve fibre damage in type 2 diabetes. The present data are not in accordance with previous studies that demonstrated the divergent development of autonomic and peripheral somatic neuropathies, which suggests different pathophysiological mechanisms [25, 26]. Differing results between our study and previously described studies can be explained by (a) different study designs, follow-up studies, and differences in inclusion criteria (Toyry included only newly diagnosed noninsulin-dependent diabetic patients), (b) different methods of evaluation of somatic dysfunction, such as using only clinical data (as in the Tentolouris study), without electrophysiological investigation. This approach could result in a higher sensitivity and accuracy of EMG for the diagnosis of peripheral neuropathy.

The basic mechanisms underlying damage to the somatic and autonomic nerves may be similar, and there are likely similar different susceptibilities of the autonomic (small and mostly unmyelinated) and peripheral somatic (large and myelinated) nerve fibres to hyperglycaemia.

This study revealed a significant correlation between CAN and glycaemic control, duration of diabetes, systolic blood pressure, lipid profile, BMI, and WHR. These findings emphasis the role of insulin resistance not only in the aetiology of the metabolic syndrome but also as a determinant of cardiac autonomic dysfunction.

The UKPDS study confirmed the findings of previous trials at the “evidence based medicine” level regarding neuropathy complicating type 2 diabetes. Efficient metabolic control can reduce the prevalence of microvascular complications, including neuropathy [6]. Our data are in agreement with this large study, suggesting that poor glycaemic control is correlated with the severity of cardiac autonomic dysfunction.

In the present study, hyperglycaemia was the main etiological factor responsible for nerve damage, but several studies have shown that other factors influence the progression of neuropathy in diabetes. The VA Cooperative Study results, for instance, established that there was no difference in the prevalence of neuropathy in diabetic patients with standard versus intensive glycaemic control [27]. In another study, the density of myelinated fibres in the sural nerve in patients with mild to moderate diabetes was associated only with elevated triglyceride levels, independent of glycaemic control or diabetes duration [28]. This finding supports dyslipidaemia as a possible accelerator of nerve damage in conjunction with hyperglycaemia. Our results are in agreement with these data because higher levels of cholesterol and triglycerides were associated with the presence of CAN, and higher cholesterol levels increased the risk of CAN in type 2 diabetes.

Obesity has been long recognised as a major risk factor for chronic disorders, particularly type 2 diabetes and cardiovascular disease. An association of obesity with CAN has been observed by some authors [29, 30]. The regional distribution of body fat is a relevant factor in the modulation of the health hazards of obesity. A preferential accumulation of body fat in the abdominal region, for instance, is associated with an increased risk of developing type 2 diabetes and atherosclerosis. Central body fat distribution (android distribution) was more closely associated with diabetes and atherosclerosis than with gynoid obesity. Our study showed a significant relationship between BMI and central fat distribution according to the WHR and the severity of CAN. These findings are in agreement with reports from the Finnish Diabetes Prevention study, where the degree of CAN was associated with higher triglyceride levels and higher waist circumference, both of which are features of metabolic syndrome [31]. Our results are only partially in accordance with a previous study performed by Valensi [9], who reported an association between impairments in autonomic function with increased weight but not with the distribution of obesity.

We found a significant correlation between the treatment modality and CAN. Patients treated with insulin were at higher risk for CAN, most likely due to poorer glycaemic control prior to the initiation of insulin treatment.

Diabetic macroangiopathy, manifested by atherosclerosis of the coronary arteries, cerebral arteries, and large arteries of the lower extremities, is the major cause of mortality and significant morbidity. IMT is a predictive indicator for the progression of cardiovascular disease, as coronary atherosclerosis development has been demonstrated to be “in the mirror” with carotid artery, and the IMT is used as an indicator for atherosclerosis [32]. Type 2 diabetic patients are known to have a significantly enhanced cardiovascular risk, and IMT is an accurate method for evaluating this risk when used in conjunction with other biochemical and constitutional parameters. In this study, we found a significant relationship between IMT values and the severity of CAN. This finding is in accordance with a previous study [33]. We also found a close association between the severity of CAN and the ABI, a marker of macroangiopathy.

In our study, we demonstrated that CAN is associated with modifiable factors such as obesity, central fat distribution, high systolic blood pressure, dyslipidaemia, smoking, and poor glycaemic control. We confirmed the existence of associations between CAN and other microvascular complications in type 2 diabetes, in particular, peripheral neuropathy and retinopathy. Importantly, we demonstrated a close association between CAN and macroangiopathic complications (ABI and IMT).

In conclusion, it is essential that risk factors associated with the progression and development of CAN be detected and treated at an early stage to further reduce morbidity and mortality. Considering all microvascular and macrovascular complications that are interrelated may facilitate early detection, early intervention, and optimum metabolic control, while eliminating other risk factors to prevent or mitigate chronic diabetic complications.

## Figures and Tables

**Table 1:** Normal, borderline, and abnormal values of cardiac autonomic function tests.

Method	Normal	Borderline	Abnormal
Tests for investigation of parasympathetic function			
(1) HR variation (R-R interval variation) during deep breathing (max-min HR) (beats/min)	>15	11–14	<10
(2) HR response to Valsalva manoeuvre (Valsalva ratio)	≥1.21	—	≤1.20
(3) HR response to standing up (30 : 15 ratio)	≥1.04	1.01–1.03	≤1.00
Tests for the investigation of sympathetic function			
(1) BP response to standing (fall in SBP) (mmHg)	≤10	11–29	≥30
(2) BP response to sustained handgrip (rise in DBP) (mmHg)	≥16	11–15	≤10

HR: heart rate, BP: blood pressure, SBP: systolic blood pressure, and DBP: diastolic blood pressure.

**Table 2:** Clinical and paraclinical characteristics of patients according to the presence of CAN.

Variable	CAN−	CAN+	*P* value
nr (%)	91 (61.1)	58 (38.9)	
Male/female, nr (%)	43 (47.2)/48 (52.8)	26 (44.8)/32 (55.2)	0.90
Age (years)	57.6 ± 8.6	59.4 ± 7.9	0.19
Age diabetes dg. (years)	53.3 ± 8.8	44.5 ± 10.1	0.0001
Diabetes duration (years)	4 (0–15)	12.5 (5–37)	0.0001
Duration of diabetes			
At onset	17 (18.7)	0 (0)	0.002
<5 years, nr (%)	45 (49.5)	1 (1.9)	0.0001
5–10 years, nr (%)	20 (21.9)	19 (32.7)	0.20
11–15 years, nr (%)	7 (7.7)	19 (32.7)	0.0002
>15 years, nr (%)	1 (1.2)	19 (32.7)	0.0001
Body mass index (kg/m^2^)	29.5 ± 5.1	32.9 ± 5.2	0.0001
SBP (mmHg)	140.8 ± 18.1	147.2 ± 19.9	0.04
DBP (mmHg)	81.2 ± 9.2	81.6 ± 10.4	0.79
HT (yes), nr (%)	57 (62.6 %)	42 (82.7)	0.01
SCORE*	4 (0–22)	6.5 (0–22)	0.0002
Exsmokers, nr (%)	4 (4.4)	1 (1.7)	0.66
Current smokers (yes), nr (%)	31 (34.1)	31 (53.5)	0.02
Nonsmokers, nr (%)	56 (61.5)	26 (44.8)	0.0001
<20 cigarettes/day, nr (%)	16 (51.6)	14 (45.2)	0.8
>20 cigarettes/day, nr (%)	15 (48.4)	17 (54.8)	0.8
Cholesterol (mg%)	204.3 ± 49.6	231.4 ± 47.8	0.001
Triglycerides (mg%)*	152.0 (60.0–996.0)	197.0 (95.0–1100.0)	0.001
HbA1c	7.9 ± 1.4	9.1 ± 1.2	0.0001
FPG (mg%)	175.9 ± 69.3	201.4 ± 55.4	0.02
eGFR (mL/min per 1.73 m^2^)	85.7 ± 17.2	59.7 ± 14.8	0.0001
OAD, nr (%)	35 (38.5)	10 (17.1)	0.009
OAD+INS, nr (%)	16 (17.6)	15 (25.8)	0.32
INSULIN, nr (%)	40 (43.9)	33 (56.9)	0.16
PNP			
Clinical, nr (%)	14 (15.4)	49 (84.5)	0.0001
Subclinical, nr (%)	38 (41.7)	5 (8.6)	0.0001
No PNP, nr (%)	39 (42.9)	4 (6.9)	0.0001
Retinopathy			
Proliferative, nr (%)	1 (1.1)	9 (15.5)	0.002
Preproliferative, nr (%)	10 (11.0)	33 (56.9)	0.0001
No retinopathy, nr (%)	80 (87.9)	16 (27.6)	0.0001
ABI*	1.02 (0.78–1.14)	0.86 (0.75–1.35)	0.0001
QTC	412.3 ± 27.7	447.4 ± 26.2	0.0001
IMT	0.83 ± 0.17	1.03 ± 0.15	0.0001
Waist-hip female	0.84 ± 0.07	0.9 ± 0.06	0.002
Waist-hip male	0.90 ± 0.06	0.97 ± 0.06	0.005

Data are shown as mean ± SD or as nr (%).

*Median, range.

CAN: cardiovascular autonomic neuropathy, SBP: systolic blood pressure, DBP: diastolic blood pressure, HT: hypertension, FPG: fasting plasma glucose, SCORE: systematic coronary risk evaluation, HbA1c: glycosylated hemoglobin, eGFR: estimated glomerular filtration rate, OAD: oral antidiabetics, INS: insulin, PNP: peripheral neuropathy, ABI: ankle-brachial index, IMT: intima-media thickness, and QTC: corrected QT interval.

**Table 3:** Univariate logistic regression analysis (CAN+, CAN− patient groups).

Variable	Odds ratio	95% CI	*P* value
Male/Female	0.90	0.4682 to 1.7569	0.772
Age (years)	1.02	0.9864 to 1.0688	0.19
Age diabetes dg. (years)	0.89	0.8571 to 0.9402	<0,0001
Diabetes duration (years)	1.6797	1.4278 to 1.9760	0.0001
Body mass index (kg/m^2^)	1.13	1.0588 to 1.2139	0.0003
SBP (mmHg)	1.01	1.0000 to 1.0360	0.05
DBP (mmHg)	1.0046	0.9710 to 1.0394	0.78
HT (yes)	1.56	0.7656 to 3.2024	0.21
SCORE	1.12	1.0425 to 1.2069	0.002
Smoking (yes versus no)	2.22	1.1327 to 4.3598	0.02
Severity of smoking (>20 versus <20 cigarettes/day)	1.29	0.4773 to 3.5148	0.6115
Cholesterol (mg%)	1.01	1.0041 to 1.0189	0.0023
Triglycerides (mg%)	1.0024	0.9999 to 1.0049	0.0633
HbA1c	1.83	1.3826 to 2.4302	<0.0001
Treatment of diabetes	3.0000	1.3458 to 6.6873	0.0072
FPG (mg%)	1.0061	1.0008 to 1.0115	0.02
eGFR (mL/min per 1.73 m^2^)	0.90	0.8808 to 0.9376	<0.0001
ABI	0.0046	0.0002 to 0.0944	0.0005
QTC	1.05	1.0341 to 1.0728	<0.0001
IMT	12.48	8.21 to 18.32	<0.0001
Retinopathy (yes versus no)	16.5306	2.0342 to 134.3347	0.008
Peripheral neuropathy (yes versus no)	29.9444	12.0439 to 74.4503	0.0001

SBP: systolic blood pressure, DBP: diastolic blood pressure, HT: hypertension, SCORE: systematic coronary risk evaluation, HbA1c: glycosylated hemoglobin, FPG: fasting plasma glucose, eGFR: estimated glomerular filtration rate, ABI: ankle-brachial index, IMT: intima-media thickness, and QTC: corrected QT interval.

**Table 4:** Multivariate logistic regression analysis.

	Odds ratio	95% CI	*P* value
Model 1			
Male/female	1.80	0.55 to 5.84	0.32
Age (years)	1.48	1.21 to 2.12	<0.0001
Diabetes duration (years)	1.74	1.46 to 2.07	<0.0001
Model 2			
Cholesterol (mg%)	1.009	1.0007 to 1.0182	0.03
Triglycerides (mg%)	0.99	0.9963 to 1.0022	0.61
HbA1c	1.88	1.3101 to 2.7202	0.0007
FPG (mg%)	0.99	0.9899 to 1.0041	0.40
Model 3			
Retinopathy (yes versus no)	4.14	0.3942 to 43.6795	0.23
Peripheral neuropathy (yes versus no)	15.47	5.1626 to 46.3864	<0.0001
IMT	17.84	0.3795 to 838.9339	0.14
QTC	1.03	1.0074 to 1.0581	0.01
ABI	0.29	0.0052 to 16.2428	0.54
Model 4			
Smoking (yes versus no)	2.21	1.0859 to 4.5307	0.02
Body mass index (kg/m^2^)	1.12	1.0530 to 1.2100	0.0006
HT	1.43	0.6713 to 3.0747	0.35

HbA1c: glycosylated hemoglobin, FPG: fasting plasma glucose, IMT: intima-media thickness, QTC: corrected QT interval, ABI: ankle-brachial index, and HT: hypertension.
